# Crowdsourcing Knowledge Discovery and Innovations in Medicine

**DOI:** 10.2196/jmir.3761

**Published:** 2014-09-19

**Authors:** Leo Anthony Celi, Andrea Ippolito, Robert A Montgomery, Christopher Moses, David J Stone

**Affiliations:** ^1^Institute for Medical Engineering and ScienceLaboratory of Computational PhysiologyMassachusetts Institute of TechnologyCambridge, MAUnited States; ^2^Beth Israel Deaconess Medical CenterDivision of Pulmonary, Critical Care and Sleep MedicineHarvard Medical SchoolBoston, MAUnited States; ^3^Engineering Systems DivisionMassachusetts Institute of TechnologyCambridge, MAUnited States; ^4^Beth Israel Deaconess Medical CenterDepartment of MedicineHarvard Medical SchoolBoston, MAUnited States; ^5^Smart Scheduling, Inc.Cambridge, MAUnited States; ^6^UVA Center for Wireless HealthDepartments of Anesthesiology and NeurosurgeryUniversity of Virginia School of MedicineCharlottesville, VAUnited States

**Keywords:** knowledge discovery, crowdsourcing, innovation, hackathon

## Abstract

Clinicians face difficult treatment decisions in contexts that are not well addressed by available evidence as formulated based on research. The digitization of medicine provides an opportunity for clinicians to collaborate with researchers and data scientists on solutions to previously ambiguous and seemingly insolvable questions. But these groups tend to work in isolated environments, and do not communicate or interact effectively. Clinicians are typically buried in the weeds and exigencies of daily practice such that they do not recognize or act on ways to improve knowledge discovery. Researchers may not be able to identify the gaps in clinical knowledge. For data scientists, the main challenge is discerning what is relevant in a domain that is both unfamiliar and complex. Each type of domain expert can contribute skills unavailable to the other groups. “Health hackathons” and “data marathons”, in which diverse participants work together, can leverage the current ready availability of digital data to discover new knowledge. Utilizing the complementary skills and expertise of these talented, but functionally divided groups, innovations are formulated at the systems level. As a result, the knowledge discovery process is simultaneously democratized and improved, real problems are solved, cross-disciplinary collaboration is supported, and innovations are enabled.

## Addressing the Knowledge Gaps in Medicine

On October 30th, 1948, Austin Bradford Hill and his colleagues at England’s Medical Research Council published “Streptomycin treatment of pulmonary tuberculosis” [1]. Using the power of a coin flip (or in this case, a random draw of an envelope), Hill was able to remove selection bias, revealing the clearest possible picture of causality then available. With this simple addition, he established the basic framework of a randomized controlled trial (RCT), a new standard for guiding evidenced-based medicine. In the years since, RCTs have upended much of clinical practice, allowing the medical field to organize into a system that creates and propagates new knowledge. Yet 65 years after the first RCT, only 10-20% of medical decisions are based on evidence [2]. And, as target populations subdivide along permutations of chronic morbid conditions and countless genetic polymorphisms, as diagnostic tools become more personalized, and as therapeutic options expand beyond the evaluation of individual drugs and devices to encompass the health care delivery network itself, it is increasingly apparent that RCTs cannot scale to match the exponential growth of medical complexity. While there are efforts underway to reduce the waste, cost, and difficulty of conducting research, clinicians and patients alike are currently left coping with a system of unacceptable ambiguity [3-6].

When faced with complex diagnostic or treatment uncertainties, patient and provider alike face several dilemmas. Combinations of diagnostic and therapeutic quandaries create the need for difficult decisions that reside in and are strongly affected by the contexts of individual patient factors and practice settings. One must determine when to probe more deeply and when to back off and observe without intervention. It is difficult, and perhaps a bit embarrassing to the medical profession, to attempt to further involve patients in the decision-making process based on current levels of uncertainty. It is not unlikely that during any clinical interaction one or more questions or problems will arise that cannot be fully addressed due to incomplete translation of research findings or clinical literature to the bedside, but more commonly, due to the incomplete state of medical knowledge.

Much of medical education consists of gaining skill and confidence not only in navigating but also in subsequently guiding others through this trail of ambiguity. One of the key objectives of the research enterprise is driving this ambiguity down so that practice is based more thoroughly on evidence. With the near ubiquitous implementation of digital documentation, we have the potential capability of answering more of these currently unresolved questions and transferring these answers into real-time workflows.

## The Full-Time Clinician and Knowledge Discovery

Ideally, from the vast amount of electronic data we already have created and further generate every day, frontline providers should be better empowered to answer the tough questions that pertain to individual patients. Better information should allow clinicians to make better decisions with a more robust element of patient involvement. However, there are real but surmountable barriers to such an approach. The condensed version of the answer is that we need more data-savvy participants as well as more carefully engineered software applications at the core of the clinical data analytic process. Clinicians should not have to become data scientists, but an appropriate awareness and understanding of basic data issues is fast becoming an important element of clinical practice. This does not represent the stumbling block for the current and upcoming generations of medical students (who have grown up in digital environments) that it may represent for older, sometimes resistant clinicians.

It would be unrealistic to expect clinicians to conduct queries of clinical data to generate evidence/data-driven decisions for each patient. This is especially so given the current lack of a technological infrastructure that would allow frontline providers to pose such questions at the point of care in real or near-real time. This reveals a critical fragility in our current knowledge generating system: the divide between the roles of researcher, data systems engineer, and clinician. This separation is detrimental—for researchers, it can make it difficult to identify knowledge gaps in clinical decision making. For engineers, the problems include identifying what is important in a foreign and complex domain, working within non-interoperable, proprietary silos, and the difficulties involved in creating effective and user-friendly clinical information systems. And for practitioners, it diminishes a sense of connection to scientific investigation and disengages busy clinicians from constructive inquiry and participation in their own clinical data systems. Overall, these systematic flaws restrict the raw number of people, ideas, and innovations that are available to address and solve the myriad problems encountered in the day-to-day care of patients.

## Democratizing Research I: Crowdsourcing and Open Data

While recognizing the challenge, we believe that it is important to find ways to democratize or “crowdsource” research. The term “crowdsourcing” was first introduced in 2005 by Jeff Howe and Mark Robinson, editors of Wired magazine, after conversations about how businesses were using the Internet to outsource work to individuals [7].

Simply defined, crowdsourcing represents the act of a company or institution taking a function once performed by employees and outsourcing it to an undefined (and generally large) network of people in the form of an open call. This can take the form of peer-production (when the job is performed collaboratively), but is also often undertaken by sole individuals. The crucial prerequisite is the use of the open call format and the large network of potential laborers.

Crowdsourcing knowledge discovery in medicine can be vertically approached by lowering the barriers of participation to frontline providers and horizontally approached by extending an input role to non-traditional but interested contributors such as patients themselves. When applied to innovations in general, this process would permit people interacting with the medical system to develop exactly what they want, rather than relying on manufacturers to act as their (often very imperfect) agents. Moreover, individual users should not have to build everything from scratch or query a database on their own: they benefit from collaborating with those who have the skillset that they lack or building on solutions developed by and freely shared by others.

Embedding user-driven research and development into communities can create connections that accelerate and enhance the innovation process, increasing the speed and effectiveness of the dissemination of a solution or new knowledge [8]. This concept has been well documented in other industries. For instance, the Defense Advanced Research Projects Agency originally developed ARPAnet to create a network of researchers and defense contractors to accelerate the exchange of software and data—this evolved into what we know as the Internet [9]. Linus Torvalds developed LINUX in 1991 as a free and open-source operating system that enabled anyone (albeit, with the requisite skill set) to contribute to its development [10].

Although medicine brings a unique set of challenges to systematic change and improvement, a more democratized approach is needed to support and optimize such processes. There have been numerous projects in the health field that have used crowdsourcing as methodology, including the three that we cite here. “FoodSwitch” is a mobile phone app that provides consumers with nutrition information obtained from users through a crowdsourcing function integrated within the app [11]. In another paper, Brown and colleagues described a method to distribute evaluation of scientific literature, a time-consuming endeavor that requires hours of coding and rating, across a large group through online crowdsourcing using Amazon’s “Mechanical Turk” [12]. Finally, Good and company developed and evaluated an online game called “The Cure”, which captured information from players regarding genes for use as predictors of breast cancer survival [13]. Their group demonstrated that crowdsourcing games can be developed as a means to address problems involving domain knowledge.

The crowdsourcing and open data movements present an opportunity to involve frontline providers and patients in accelerating innovation, including knowledge creation. Not unlike dispersed crowdsourcing networks, “hackathons” and data marathons provide opportunities for those at the front line of care, who are most familiar with the pain points within and the information gaps that plague day-to-day practice, to contribute to the much needed health care transformation. Knowledge discovery and innovations have been activities traditionally limited to doctors who have devoted their careers to research in academia or consulting in industry, or those who have given up clinical medicine. Funding to pursue academic research and opportunities to direct biotech research are seldom available to clinicians who spend most of their time in practice. Research is deemed exclusive to those with training in experimental methodologies and/or data analytics, while an additional degree in business is favored in biomedical entrepreneurship or consulting. As a result, there is always a level of disconnect between the foci of research and innovation, and solutions that will truly improve care delivery and health outcomes. In addition, practicing clinicians are almost always exhausted by the daily grind, frustrated by the inefficiencies of the health care system, and very seldom given the time, the opportunity, and the incentive to step back and address systems-level problems, including knowledge gaps in the practice of medicine.

## Democratizing Research II: Hackathons and Data Marathons

Traditionally, hackathons are 24- to 48-hour events at the front end of the innovation process that provide an accessible forum to pitch complex, difficult problems and develop initial solutions and prototypes in a quick, iterative manner. Health care hackathons and data marathons provide opportunities for providers to collaborate with engineers or data scientists and quickly make an impact by offering the clinical perspective around problems or information gaps. Similarly, these events present a great forum for engineers and data scientists to gain access to real problems in medicine and to engage with stakeholders who have expert domain knowledge. By teaming up engineers and data scientists with clinicians, the hackathons and data marathons provide the non-clinicians a rare opportunity to transform health care. And while a truly novel discovery or a fully functioning solution is a rare outcome at the end of the event, we believe health care-focused hackathons and data marathons enable crowdsourcing of valuable and diverse points of view as well as creating new personal connections that will form the basis for productive longer-term collaborations.

The authors of this article have helped organize numerous hackathons and data marathons that have brought together engineers, data scientists, and clinicians (including nurses, pharmacists, and other allied health personnel) to address problems and questions identified during routine clinical practice, including the Critical Data Marathon held at the Massachusetts Institute of Technology (MIT) in January 2014 (see Multimedia Appendix 1). To date, the MIT Hacking Medicine has organized 17 events in the United States, India, Uganda, and Spain with a diverse set of partners including the Laboratory of Computational Physiology and Sana at MIT, the Consortium for Affordable Medical Technologies (CAMTech) at the Massachusetts General Hospital (MGH), and Brigham and Women’s Hospital. These events have resulted in over 600 innovative ideas and over 250 teams that have developed prototypes, launched products, and/or published papers. In addition, a number of other organizations and initiatives are contributing to this hackathon movement in the health care community, including Health 2.0, Hacking Health, and the MedStar Institute for Innovation.

An example of the type of innovation that results from the collaboration fostered during hackathons is the Augmented Infant Resuscitator (AIR) [14]. Dr Data Santorino, a pediatrician in Uganda and researcher at Mbarara University of Science and Technology, presented the problem of newborn deaths from improper resuscitation techniques seen in low-income countries. At one of the hackathons held at MGH, he teamed up with an engineer from MIT, another clinician from MGH, and a business entrepreneur, and developed the prototype for AIR. The project has since gained considerable investment and the device is currently in field trials in Uganda.

For the MIT Critical Data Marathon held in January 2014, participants worked directly on a large, open-access clinical database called MIMIC, short for Multi-parameter Intelligent Monitoring in Intensive Care [15]. The database is the creation of a public-private partnership between the Beth Israel Deaconess Medical Center (BIDMC) in Boston, MIT, and Philips Healthcare. Committed to archiving all available data from the intensive care units at BIDMC—rather than a subset considered relevant at the time—researchers subsequently de-identify and publish the database for easy access at no cost. We believe that providing the data to as many people as possible is the best way to unlock the functionally cryptic information in electronic health records for translation into valuable information. MIMIC has attracted both clinicians and data scientists who have partnered on many outcome studies that have included examinations of practice variability, the heterogeneity of treatment effects, cost analyses, and predictive modeling, among others. Clinicians who have not typically engaged in research but who possess a deep understanding of the information gaps as well as the elements involved in medical practice are now empowered to contribute to and become part of a data-driven learning system.

## Achievable Benefits

We have previously commented on how open data and crowdsourcing may address but at the same time potentially augment the problem of unreliable and wasteful research [16]. The issue stems from the irreproducibility of what gets published and the inability of researchers to know what is not getting published. Our group organized a conference in conjunction with the data marathon held in January 2014 to address these concerns [16]. Thought leaders from academia, government, and industry across disciplines gathered and discussed the pitfalls and challenges of the data revolution sweeping health care. The consensus seemed to be that success will require a systematized and fully transparent data interrogation, where data and methods are freely shared among different groups of investigators addressing the same or similar questions. The added accuracy of the scientific findings is only one of the benefits of the systematization of the open data movement. Another will be the opportunity afforded to individuals of every educational level and area of expertise to contribute to science.

The Critical Data Marathon is one example of the realization of the goal of MIMIC: the democratization of medical research and crowdsourcing of knowledge discovery. We witnessed clinicians excitedly pairing with data scientists to work together in translating and parsing their questions into study designs and methodologies; nurses and doctors providing data scientists with essential but nuanced clinical contexts; and even architects and designers assisting in the visualization of findings. By engaging practicing clinicians as well as future ones, that is, medical students, we enable them to contribute to innovation at the systems level. Open questions remain on how to scale data sets and the infrastructure supporting the use of these data sets. But despite such challenges, the crowdsourcing movement is slowly transforming the medical culture into one where there is no divide between research and practice. These hackathons and data marathons provide a platform for frontline health care workers to create solutions to the problems in which they are immersed, democratize innovations and research in health care by engaging those who may not see themselves as academics or entrepreneurs, and harness the power of cross-disciplinary collaboration at a much larger scale.

## Multimedia Appendix 1

Inaugural MIT Critical Data Marathon, January 3-5, 2014. Photos courtesy of Andrew Zimolzak.

## Abbreviations

AIR: Augmented Infant Resuscitator
BIDMC: Beth Israel Deaconess Medical Center
MGH: Massachusetts General Hospital
MIMIC: Multi-parameter Intelligent Monitoring in Intensive Care
MIT: Massachusetts Institute of Technology
RCT: randomized controlled trial
