# Supplementary figures and images for: Crowdsourcing Knowledge Discovery and Innovations in Medicine
Source: J Med Internet Res. 2014 Sep 19;16(9):e216. doi: 10.2196/jmir.3761 (PMC4180345; doi:10.2196/jmir.3761)

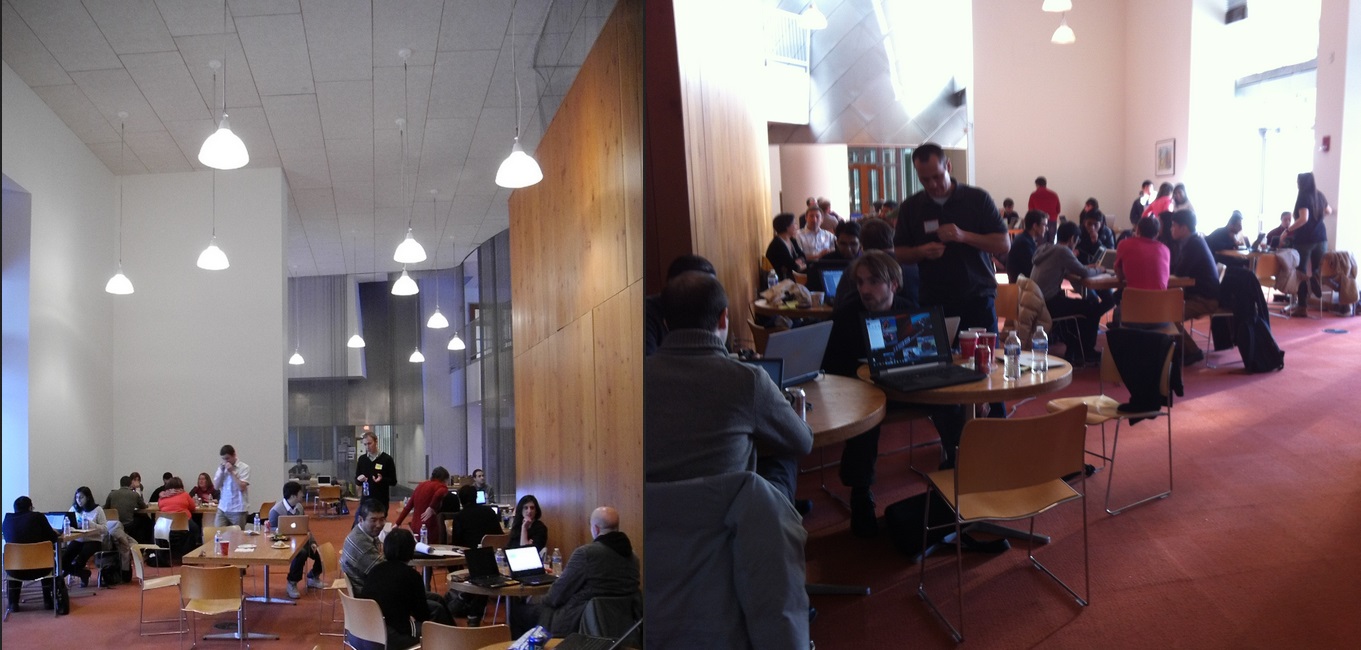

Supplement: Supplementary file 1 [file jmir_v16i9e216_app1.jpg]
